# Recurring Necrotic Enteritis Outbreaks in Commercial Broiler Chicken Flocks Strongly Influence Toxin Gene Carriage and Species Richness in the Resident *Clostridium perfringens* Population

**DOI:** 10.3389/fmicb.2017.00881

**Published:** 2017-05-17

**Authors:** Marie-Lou Gaucher, Gabriel G. Perron, Julie Arsenault, Ann Letellier, Martine Boulianne, Sylvain Quessy

**Affiliations:** ^1^Research Chair in Meat Safety, Département de Pathologie et Microbiologie, Faculté de Médecine Vétérinaire, Université de Montréal, Saint-HyacintheQC, Canada; ^2^Swine and Poultry Infectious Diseases Research Center, Département de Pathologie et Microbiologie Vétérinaire, Faculté de Médecine Vétérinaire, Université de Montréal, Saint-HyacintheQC, Canada; ^3^Chair in Poultry Research, Département de Sciences Cliniques, Faculté de Médecine Vétérinaire, Université de Montréal, Saint-HyacintheQC, Canada; ^4^Reem-Kayden Center for Science and Computation, Department of Biology, Bard College, Annandale-On-HudsonNY, United States

**Keywords:** commercial broiler chickens, drug-free program, necrotic enteritis, *Clostridium perfringens*, richness

## Abstract

Extensive use of antibiotic growth promoters (AGPs) in food animals has been questioned due to the globally increasing problem of antibiotic resistance. For the poultry industry, digestive health management following AGP withdrawal in Europe has been a challenge, especially the control of necrotic enteritis. Much research work has focused on gut health in commercial broiler chicken husbandry. Understanding the behavior of *Clostridium perfringens* in its ecological niche, the poultry barn, is key to a sustainable and cost-effective production in the absence of AGPs. Using polymerase chain reaction and pulsed-field gel electrophoresis, we evaluated how the *C. perfringens* population evolved in drug-free commercial broiler chicken farms, either healthy or affected with recurring clinical necrotic enteritis outbreaks, over a 14-month period. We show that a high genotypic richness was associated with an increased risk of clinical necrotic enteritis. Also, necrotic enteritis-affected farms had a significant reduction of *C. perfringens* genotypic richness over time, an increase in the proportion of *C. perfringens* strains harboring the *cpb2* gene, the *netB* gene, or both. Thus, necrotic enteritis occurrence is correlated with the presence of an initial highly diverse *C. perfringens* population, increasing the opportunity for the selective sweep of particularly virulent genotypes. Disease outbreaks also appear to largely influence the evolution of this bacterial species in poultry farms over time.

## Introduction

To meet the constantly growing demand for poultry meat, high production standards must be met to ensure optimum feed efficiency, weight gain, and subsequent meat yield (Daniel et al., 2011). A healthy and functional digestive tract in commercially raised broiler chickens is important for promoting such conditions (Van Immerseel et al., 2004). Antibiotic growth promoters (AGPs) and anticoccidial drugs have long been used to help balance the precarious intestinal health of broiler chickens and to prevent a disease state under intensive commercial production conditions (Porter, 1998; Daniel et al., 2011). However, the large-scale use of antimicrobials in animal production is no longer considered wise due to concerns about food-producing animals acting as a reservoir for antibiotic resistant pathogenic bacteria transmissible to humans (Perron et al., 2007; Seal et al., 2013). Unfortunately, antimicrobial withdrawal has a major impact on gut health in intensively reared broiler chickens (Timbermont et al., 2011). Indeed, following the European ban on AGPs in 1999, production performances were negatively impacted, which correlated with an increased incidence of digestive problems in poultry flocks, such as necrotic enteritis (NE) (Van Immerseel et al., 2009). These regulatory restrictions forced the poultry industry to look for sustainable alternatives to preserve gut health and to minimize animal loss (Yegani and Korver, 2008). NE is a disease caused by *Clostridium perfringens* and impacts the economic, welfare, as well as the food safety aspects of the broiler chicken production (Van Immerseel et al., 2004, 2016). Until now, no alternative strategy has been completely cost-effective in replacing AGPs. This situation makes any research efforts aiming at better understanding the role of *C.* *perfringens* in necrotic enteritis highly relevant (Huyghebaert et al., 2011; Smith, 2011).

A previous field study was conducted by our group and compared two different rearing protocols for commercial broiler chickens (Gaucher et al., 2015). We compared a conventional protocol where birds were fed a diet containing antimicrobials and a drug-free program where antimicrobials were replaced by a combination of alternative strategies, including essential oil-based products added to poultry feed, acidification of the drinking water, anticoccidial vaccination at the hatchery, and improved brooding conditions. For all farms participating in the drug-free program, nutritional guidelines pertaining to feed composition were provided (Gaucher et al., 2015). We found that the drug-free program was associated with an increased incidence of clinical NE outbreaks (Gaucher et al., 2015). Indeed, of the eight participating farms, recurring NE was observed in the same two farms over the course of our study, even if the drug-free program was uniformly applied to all. This trend has also been previously reported by Smith (2011). Thus, it is important to better characterize the *C. perfringens* community found in these healthy and NE-affected farms and to test how *C. perfringens* population richness correlates with their health status (Dumas et al., 2011).

Here we compare the *C. perfringens* richness and virulence genes carriage initially found in these healthy and NE-affected farms, and track how the bacterial population changed over time according to flock health status.

## Materials and Methods

### Sample Selection

*Clostridium perfringens* strains were recovered from fecal samples collected at the end of the rearing cycle during visits performed in commercially raised drug-free broiler chicken flocks. These farms participated in a previous study conducted by our group from May 2011 to July 2012 (Gaucher et al., 2015). All *C. perfringens* strains used in the current study were recovered only from farms participating in the drug-free program. Among the eight participating drug-free farms of the field study, only *C. perfringens* strains isolated from three drug-free healthy farms and from two drug-free farms affected with recurring clinical NE outbreaks (i.e., NE outbreaks observed within every flock over the study period) were included in the current work. The three other participating farms of the aforementioned field study were excluded from the current work as they had been categorized as farms experiencing the sub-clinical form of the disease (Gaucher et al., 2015). Data pertaining to farm performance parameters that were used for the selection of the flocks studied included here are given in **Table 1**. For the sampled farms, the first two (initial time point, periods 1 and 2) and the last two (final time point, periods 6 and 7) production periods of the field trial were considered for molecular analysis. For the sampled farms, both sampling time points were approximately 6 months apart, and no in-feed antibiotics were used on the farm during those initial and final time points. Nevertheless, because of the severity of a specific NE outbreak that occurred on both NE-affected farms during the middle period (period 4) of the field study, an antibiotic treatment (penicillin) was administered to sick birds via their drinking water. This treatment was used only once on each affected farm. However, no *C. perfringens* strains recovered from this fourth production period were included in the current study. Because *C. perfringens* strains were not recovered from all production periods and time points, and because one sick farm withdrew prematurely from the project, this study includes a total of nine healthy (from 12) and seven diseased (from eight) flocks (**Table 1**). For fecal sample collection, each floor was divided into four longitudinal parallel rows corresponding to the walking path of the sampler. Up to ten fecal samples were collected. Thus, for every pen, approximately 10 g of a pooled sample of fresh feces were randomly collected on the floor surface and put into a Whirlpack^®^ bag containing *Brucella* broth freezing media (Becton, Dickinson and Company, Sparks, MD, United States) with 30% glycerol (Fisher Scientific, Bridgewater, NJ, United States). Bags were kept on ice and brought back to the laboratory where microbiological analysis was done.

**Table 1:** Estimates (standard deviation) of performance parameters of all drug-free flocks, which served as a basis for selecting production periods included in the current study.

Performance parameters	Diseased (*n* = 11 flocks from 2 farms)	Healthy (*n* = 21 flocks from 3 farms)
Livability (%)	97.25 (1.96)	98.56 (1.76)
Condemnations (%)	3.31 (2.58)	2.52 (1.19)
Mean live weight at slaughter (kg)	2.33 (0.15)	2.45 (0.22)
Mean daily weight gain (g/d)	61.90 (3.27)	60.86 (1.55)
Kg per square meter (kg)	25.46 (1.93)	27.88 (2.11)
Clinical necrotic enteritis (% of affected flocks)	100	0


**Table 2:** Description protectof *C. perfringens* positive flocks according to the number of *C. perfringens* isolates recovered and toxin gene carriage, per farm health status and sampling time points of the study.

Characteristics	Number of flocks	Number of isolates	% positive flocks/strains for each toxin gene							
			
			*cpa*	*cpb*	*cpb2*	*tpeL*	*netB*	*iA*	*etx*	*cpe*
**Healthy**								
Initial	4	18	100/100	0/0	75/78	0/0	50/67	0/0	0/0	0/0
Final	5	30	100/100	0/0	80/90	0/0	60/87	0/0	0/0	0/0
**Diseased**								
Initial	4	45	100/100	0/0	50/31	0/0	75/20	25/22	0/0	0/0
Final	3	41	100/100	0/0	100/100	33/2	100/100	0/0	0/0	0/0


### Isolation of *Clostridium perfringens Strains*

A direct plating isolation protocol was used. One milliliter of suspended feces was inoculated on Perfringens Agar Base media (LAB M, United Kingdom) plates containing 5% egg yolk emulsion and 400 mg/L of D-cycloserine as a selective agent (LAB M, United Kingdom). Plates were then incubated in anaerobic conditions (AnaeroGen Gas Generating System, Oxoid, Nepean, ON, Canada) at 37°C for 48 h. Bacterial growth from the last quadrant was streaked on 5% sheep blood anaerobic *Brucella* agar plates with neomycin (Oxoid, Nepean, ON, Canada), and grown under anaerobic conditions at 37°C for 48 h. All suspect colonies showing typical beta-hemolysis were selected, and their identity was confirmed by the reverse CAMP (Christie, Atkins and Munch-Peterson) test. Strains that were recovered were frozen in *Brucella* broth (Becton, Dickinson and Company, Sparks, MD, United States) freezing media containing 30% glycerol (Fisher Scientific, Bridgewater, NJ, United States) at -80°C (Chalmers et al., 2008) until further characterization.

### *Clostridium perfringens* Reference Strains

The following strains were used as positive controls in this study: *C. perfringens* type A (AHL316, positive for *plc* gene), *C. perfringens* type A (AHL311, positive for *plc* and *cpe* genes), *C. perfringens* type A (STF2003-1256, positive for *plc* and *netB* genes), *C. perfringens* type B (AHL156, positive for *plc*, *cpb* and *etx* genes), *C. perfringens* type D (AHL344, positive for *plc* and *etx* genes), and *C. perfringens* type E (AHL155, positive for *plc*, *iA*, *cpe* and *cpb2* genes).

### Toxinotyping by Multiplex PCR

All *C. perfringens* strains recovered were also screened for the presence of virulence genes encoding toxins. DNA extraction was performed according to Charlebois et al. (2012) with slight modifications for PCR optimization. Briefly, isolates were grown overnight on 5% sheep blood agar plates (Oxoid, Nepean, ON, Canada) at 37°C under anaerobic conditions (AnaeroGen Gas Generating System, Oxoid, Nepean, ON, Canada). A loopful of colonies was suspended in 100 μL of a 10% Chelex 100 solution (Bio-Rad, Mississauga, ON, Canada) and boiled for 20 min. After centrifugation (12,000 *g* for 3 min), the supernatant containing DNA was collected and used for multiplex PCR. Multiplex PCR protocols were used for the detection of the toxin-encoding genes *cpa* (alpha), *cpb* (beta), *cpb2* (ß2), *cpe* (enterotoxin), *iA* (iota), *etx* (epsilon), *netB* (NetB) and *tpeL* (TpeL). Primer sequences and reaction conditions used for the PCR assays were the same as those originally described in Keyburn et al. (2010) and Charlebois et al. (2012). PCR amplification of the genomic DNA was performed in a 25 μL reaction volume and was done using 2.5 μL of 10× PCR buffer (Invitrogen, Burlington, ON, Canada), 0.05 μM of MgCl_2_ (Invitrogen, Burlington, ON, Canada), 3 units of Platinum^®^ Taq DNA polymerase (Invitrogen, Burlington, ON, Canada), 25 μM of dNTPs (Bio Basic Inc., Markham, ON, Canada), and specific concentrations of each selected primer (Invitrogen, Burlington, ON, Canada): 4.2 μM *cpa*, 1.28 μM *cpb*, 2.72 μM *cbp2*, 0.8 μM *tpeL*, 0.9 μM *cpe*, 0.6 μM *netB*, 4 μM *iA*, and 0.7 μM *etx*. Denaturation (94°C, 60 s), annealing (55°C, 60 s), and extension (72°C, 60 s) were performed for 35 cycles. A hot start step of 2 min at 94°C and a 10 min final elongation step at 72°C were added. A 12.5 μL volume of the PCR reaction product was submitted to electrophoresis using a 1% agarose gel containing 0.01% SYBR Safe DNA gel stain (Invitrogen, Burlington, ON, Canada). A 100 bp ladder (Track It, Invitrogen, Burlington, ON, Canada) was used as a molecular weight marker.

### Genotyping by Pulsed-Field Gel Electrophoresis

Plug preparation, restriction digestion, and electrophoresis conditions were essentially performed according to the protocol previously described for *C. perfringens* by Chalmers et al. (2008). Isolates were grown at 37°C overnight on 5% sheep blood agar (Oxoid, Nepean, ON, Canada) in anaerobic conditions (AnaeroGen Gas Generating System, Oxoid, Nepean, ON, Canada). Because DNA degradation is a problem when typing *C. perfringens* strains with PFGE, a formaldehyde treatment step, as described by Gibson et al. (1994), was added to the initial protocol. Briefly, harvested colonies were suspended in a 900 μl suspension buffer (75 mM NaCl, 25 mM EDTA, pH 8) volume to a final OD of 1.25 at 600 nm. One hundred microliter of a 40% formaldehyde solution (Fisher Scientific, Bridgewater, NJ, United States) were added to the bacterial suspension. Suspensions were incubated for 1 h at room temperature. Bacterial cell washes with saline (0.85%) were then repeated three times, and a final step involving bacterial cell suspension in 500 μL saline was done. This final volume was embedded in an equal volume of 1.0% melted SeaKem Gold Agarose (Lonza, United States), poured into plug molds for solidification at 4°C for 30 min. Cell lysis was done by incubating the plugs at 37°C with gentle shaking for 5 h in a lysis buffer (10 mM Tris, 100 mM EDTA, 50 mg/ml lysozyme (Sigma-Aldrich, St. Louis, MO, United States). Plugs were rinsed for 15 min in TE buffer (10 mM Tris, 100 mM EDTA) and incubated overnight in EDTA 0.5 M, 1% sarkosyl (Sigma-Aldrich, St. Louis, MO, United States), and 2 mg/ml proteinase K (Fisher Scientific, Canada). To remove traces of proteinase K, plugs were rinsed five times for 30 min each in washing buffer (10 mM Tris, 1 mM EDTA, pH8). One plug per isolate was equilibrated in 200 μL restriction buffer (New England BioLabs, Ipswich, MA, United States) at room temperature for 20 min and then transferred into 200 μL of digestion buffer containing 100 U of SmaI restriction enzyme (Chalmers et al., 2008) (New England BioLabs Inc., Ipswich, MA, United States). Low Range PFG marker (New England BioLabs Inc., Ipswich, MA, United States) was used as a molecular weight standard. Electrophoresis was performed in TE 1% SeaKem Gold Agarose gel and ran in a 2.500 L volume of 0.5% TBE buffer containing 200 μM thiourea (Sigma-Aldrich, St. Louis, MO, United States) at 14°C for 19 h. Pulse times started at 4 s and ended at 38 s, with linear ramping and a field of 6 V/cm in a Bio-Rad CHEF II electrophoresis unit (Bio-Rad Laboratories, Hercules, CA, United States). Gels were stained with ethidium bromide, and photographs were saved as tiff files and loaded into BioNumerics software v6.0 (Applied Maths NV) for band matching. The unweighted pair group for arithmetic means (UPGMA) tree-building approach was used for band matching. A 1.5% optimization and a 2.3% position tolerance were selected, and a visual inspection step was carried out to ensure appropriate clustering of identical and different genotypes. Macrorestriction patterns were similarity compared using the Dice coefficient. A dendrogram was generated. A similarity of more than 97% was set as a limit for isolates to be considered as members of the same cluster (Gholamiandekhordi et al., 2006).

### Statistical Analyses

#### Richness Analysis

First, we compared the number of strains isolated between healthy and NE-affected flocks. A binomial-negative regression was used to model the number of isolates per flock, the outcome variable herein, with the flock health status (e.g., healthy vs. NE-affected) and sampling time (e.g., initial vs. final) as explanatory variables. A robust variance estimate was used to take into account the potential clustering within farms. The interaction between the two explanatory variables was also tested. The GENMOD procedure of SAS 9.4 was used.

Using rarefaction and extrapolation curves, we then compared the genotypic richness observed among *C. perfringens* strains according to two different parameters: farm health status (e.g., healthy vs. NE-affected) and sampling time point (e.g., initial vs. final) of the study period. Rarefaction curves are plots of the cumulative species richness as a function of the numbers of individuals sampled allowing comparison of species richness between specific environments on an equal effort basis (Gotelli and Colwell, 2001). Rarefaction and extrapolation curves with 95% confidence intervals were computed using the iNEXT package as implemented in the R programming environment (v.3.1.2) (Chao et al., 2014). Completedness curves were also computed to illustrate how efficient the sampling effort was to reveal the whole genotypic richness from the sampled farms.

#### Toxinotyping Analysis

Logistic regression was used to model the presence of *cpb2* toxin at the strain level, herein considered as the outcome variable, as a function of the presence of the *netB* gene. A robust variance estimate was used to take into account the potential clustering of strains within flocks. The GENMOD procedure of SAS 9.4 was used. No other association between toxin genes was investigated based on results pertaining to toxin gene carriage.

We then used a series of median exact tests to investigate whether the carriage of toxin genes per flock was linked to sampling time points and NE status. Four outcomes were studied and were defined as the proportion of *C. perfringens* isolates carrying the *netB* gene, *cpb2* gene, ≥2 toxin genes, and ≥3 toxin genes. Flocks for which no *C. perfringens* strains were recovered were excluded from the analyses. For each outcome, a comparison was made between healthy and diseased flocks for initial and final sampling time points. The association was also investigated between the initial and final time points of the study, and again, was performed separately for healthy and diseased barns. Since this study was intended to be exploratory in nature, no adjustment was made for the *p*-value interpretation when multiple comparison tests were performed.

## Results

### *Clostridium perfringens* Isolation and Typing

A total of 134 *C. perfringens* strains were recovered from the fecal samples collected during the first two and the last two production periods on the two NE-affected and the three healthy drug-free farms of the field study. Details about farms, production periods, strains recovered and genotypes identified are presented in **Table 3**. Isolates were submitted to multiplex toxinotyping PCR, as well as to PFGE profiling using *SmaI* restriction endonuclease. Among the 134 strains, 130 were successfully typed using *SmaI* enzyme and generated 43 distinct genotypes, each one of these comprising between seven and twelve bands. The inter-gel migration variability was taken into account and, accordingly, all PFGE gels were manually inspected and analyzed.

**Table 3:** Description of the number of isolates, genotypes identified and toxin gene carriage per flock according to farm health status and production period.

Farm id	Nb isolates	Genotypes identified		Positive isolates per flock						
			
			*netB*		*cpb2*		≥2 toxin genes		≥3 toxin genes	
						
			Nb	%	Nb	%	Nb	%	Nb	%
**Healthy flocks**										
Initial time point										
Farm 3	2	9	2	100	2	100	2	100	2	100
Farm 3	0	–	0	–	0	–	0	–	0	–
Farm 5	3	11–42	0	0	2	67	2	67	0	0
Farm 5	3	41	0	0	0	0	0	0	0	0
Farm 8	0	–	0	–	0	–	0	–	0	–
Farm 8	10	10–18–17	10	100	10	100	10	100	10	100
*Median*	*2.5*		*0*	*50*	*1*	*83*	*1*	*83*	*0*	*50*
Final time point										
Farm 3	3	1	0	0	0	0	0	0	0	0
Farm 3	1	2	0	0	1	100	1	100	0	0
Farm 5	0	–	0	–	0	–	0	–	0	–
Farm 5	10	2–7–8	10	100	10	100	10	100	10	100
Farm 8	7	3–34	7	100	7	100	7	100	7	100
Farm 8	9	34–35	9	100	9	100	9	100	9	100
*Median*	*5*		*3.5*	*100*	*4*	*100*	*4*	*100*	*3.5*	*100*
**Necrotic enteritis flocks**										
Initial time point										
Farm 4	15	20–21–25–38–39–40	1	7	0	0	1	7	0	0
Farm 4	14	27–32–33–36–37–43	1	7	0	0	11	79	0	0
Farm 7	15	13–14–15–16–22–26	7	47	13	87	13	87	7	47
Farm 7	1	30	0	0	1	100	1	100	0	0
*Median*	*14.5*		*1*	*6.9*^a^	*0.5*	*43.3*	*6*	*82.6*	*0*	*0.0*^a^
Final time point										
Farm 4	14	12–19–31	14	100	14	100	14	100	14	100
Farm 4	0	–	0	–	0	–	0	–	0	–
Farm 7	20	4–5–6–28–29	20	100	20	100	20	100	20	100
Farm 7	7	23–24	7	*100*	7	100	7	100	7	100
*Median*	*10.5*		*10.5*	*100*^b^	*10.5*	*100*	*10.5*	*100*	*10.5*	*100*^b^


*^a,b^Median values with different superscripts within a column are statistically different (*p* < 0.05, exact median test).*

### *Clostridium perfringens* Genotypic Richness

Descriptive statistics on the number of isolates per flock are presented in **Table 3**. The number of recovered isolates was 2.8-times higher (incidence ratio, *p* = 0.01) in the NE-affected flocks than in healthy flocks. No association was found between the number of strains recovered and the sampling time point (*p* = 0.28). The interaction between flock health status and sampling time point was not statistically significant (*p* = 0.19) and thus, was not kept in the model.

**FIGURE 1:**
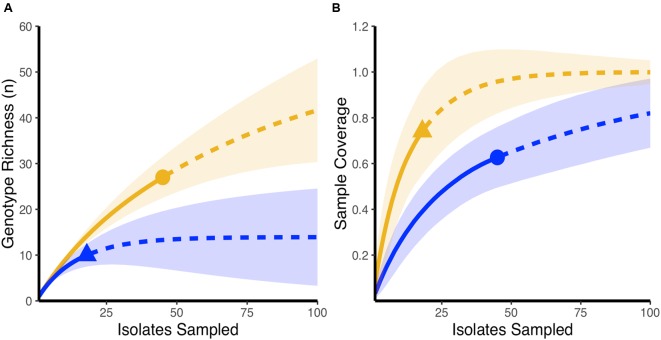
**Initial genotypic richness on healthy farms and farms that develop necrotic enteritis outbreaks over the course of the experiment.** **(A)** Rarefaction-based accumulation curves show that initial genotypic richness was higher in farms that developed necrotic enteritis (orange) when compared to farms that maintained a healthy status (blue). Solid curves show the interpolated rarefaction curve from the sampled strains, while dashed line show extrapolation to at last double the maximum sampling effort described in this study. **(B)** Completedness curves show that the observed sample number does not likely reveal the full extent of genotypic richness in farms that developed necrotic enteritis (blue) at the start of the study. In **(A,B)**, dots and triangles are observed sample number and shaded areas are 95% confidence intervals based on 100 bootstrap values.

**FIGURE 2:**
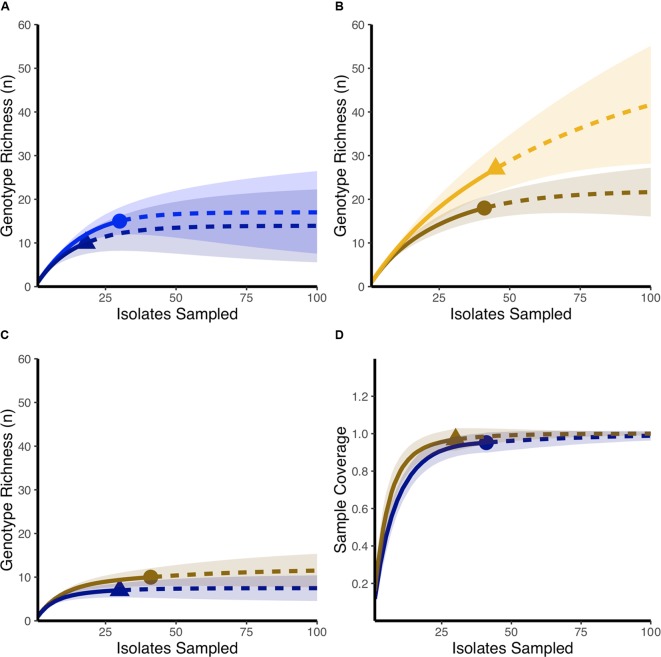
**Comparing final and initial genotypic richness in healthy farms and farms that develop necrotic enteritis outbreaks.** **(A)** Rarefaction-based accumulation curves show that the final genotypic richness in healthy farms (dark blue) was similar to genotypic richness observed as the start of the study (blue). **(B)** Rarefaction-based accumulation curves show that final genotypic richness in farms that developed necrotic enteritis (dark orange) is lower than genotypic richness observed in the same farms at the start of the study (orange). **(C)** Rarefaction-based accumulation curves show that final genotypic richness in farms that developed necrotic enteritis (dark orange) is not statistically different than genotypic richness observed in healthy farms (dark blue) at the end of the study. In **(A–C)** solid curves show the interpolated rarefaction curve from the reference sample while dashed lines show extrapolation to at last double the maximum sampling effort described in this study. **(D)** Completedness curves show that final observed sample numbers likely revealed the full extent of genotypic richness in both healthy farms (dark blue) and farms that developed necrotic enteritis (dark orange). Dots and triangles identify observed sample number and shaded areas are 95% confidence intervals based on 100 bootstrap values.

Using rarefaction curves, we found that at the start of the experiment, farms that would later be affected by NE during the study period had a higher genotypic richness compared to farms that remained healthy (**Figure 1A**). Interestingly, completedness curves indicate that the sampling procedure conducted at the start of the experiment did not reveal all the genotypes present in farms that would experience NE (**Figure 1B**), suggesting that the genotypic richness present in these farms might be even higher than estimated in the current study. At the final sampling time point, while we observed no change in the genotypic richness found in healthy farms compared to the initial sampling (**Figure 2A**), we observed a significant decrease in genotypic richness in farms that were affected by NE outbreaks (**Figure 2B**). Indeed, the genotypic richness in NE-affected farms was similar to that of healthy farms by the end of the sampling period (**Figure 2C**). This suggests that a selective sweep likely occurred in farms where genotypic richness was initially high. Completedness curves obtained for the final sampling time point showed that *C. perfringens* genotypic diversities found in healthy and NE-affected farms were fully revealed by the sampling procedure (**Figure 2D**).

### *Clostridium perfringens* Toxinotyping by Multiplex PCR

All 134 strains tested were found to be positive for the presence of the *cpa* gene, confirming *C. perfringens* identity. Results pertaining to toxinotyping results are presented in **Tables 2**, **3**. While 98% of the 88 strains carrying the *netB* gene were also found to be positive for the presence of the beta-2 toxin gene (*cpb2*), only 22% of the 46 *netB* negative *C. perfringens* strains were identified with *cpb2*. This association was statistically significant based on the logistic regression model (odds ratio = 172, *p* < 0.001). At the flock level, the median proportion of *C. perfringens* isolates carrying the *netB* gene, as well as the proportion of these strains harboring three toxin genes, were found to be significantly higher (*p* = 0.03) at the final sampling time point of the field study when compared to the initial sampling time point, but only for NE-affected farms (**Table 3**). No other associations between toxin gene presence in the studied *C. perfringens* strains and NE status or time period were identified as statistically significant.

## Discussion

This study provides new insights into the dynamics of *C. perfringens* populations in drug-free farms, according to their digestive health clinical profile: healthy vs. diseased (clinical NE-positive). Although many studies have been conducted to better describe the role of *C. perfringens* in necrotic enteritis pathogenesis, little work has been done to better characterize this microorganism population structure in commercial broiler chicken farms, where the use of antimicrobials has been discontinued (Prescott et al., 2016; Rood et al., 2016). More importantly, much remains to be explained to better understand why raising drug-free birds would be more challenging on some farms in regard to digestive health management and NE issues (Brady et al., 2010; Smith, 2011). Here, we show that farms presenting with a higher genotypic richness of *C. perfringens* were more likely to develop NE over a 14-month period. Furthermore, we found that following recurring NE outbreaks, diseased farms harbored a decreased genotypic richness, suggesting that one or more genotypes were selectively increased in frequency during the outbreaks.

In healthy and well performing farms, *C. perfringens* species richness was lower and relatively well assessed during the sampling done at the initial time point of the study, an affirmation supported by the more rapidly plateauing appearance of the rarefaction and completedness curves (Gotelli and Colwell, 2001; Hughes and Hellmann, 2005). Conversely, one unexpected result was the presence of a higher and not completely revealed genotypic richness found at the beginning of the field study in farms that would experience recurring NE outbreaks during the study (**Figure 1B**). This higher richness could have increased the likelihood of one or many virulent clones being present in the *C. perfringens* population at the start of the study. Indeed, it has been reported that a mild sub-clinical form of NE can exist even in the presence of antibiotics and more importantly, can be correlated with a larger and a more diverse *C. perfringens* population (Johansson et al., 2010; Lyhs et al., 2013). Given the absence of antibiotics, a less well-controlled *C. perfringens* population has likely played a role in the occurrence of NE outbreaks on the diseased farms of our study, as has been reported previously by others (Watkins et al., 1997; Johansson et al., 2004; Martel et al., 2004; Silva et al., 2009; Gharaibeh et al., 2010; Charlebois et al., 2012). Both antimicrobial resistance and virulence genes can contribute to the adaptation process of *C. perfringens*, and although the presence of antibiotic resistance genes in NE-causing strains was most probably not the cause of the NE outbreaks in the diseased farms of our field study, it may have contributed to it by fostering the colonization of these farms by *C. perfringens* strains harboring these plasmids (Beceiro et al., 2013). Assessing the antibiotic resistance profile of the recovered *C. perfringens* strains from these farms at the end of the study could then be interesting, as the genes encoding antibiotic resistance can be found on the same plasmids carrying the toxin genes that showed a significant increase in frequency over time in the diseased farms of our study (Harrison and Brockhurst, 2012; Palliyeguru and Rose, 2014). If that is the case, the recurring nature of NE outbreaks observed on these farms could have contributed to an increased prevalence of strains showing antibiotic resistance at the end of the experiment (Beceiro et al., 2013).

Based on rarefaction curves, the changes in *C. perfringens* richness over the study period also showed great differences between the farm clinical profiles. Interestingly, in healthy and well-performing farms, rarefaction and completedness curves showed that *C. perfringens* richness was relatively well assessed by our sampling procedure at the initial and final time points. However, only the final sampling revealed the complete species richness for the NE-affected farms; the initial sampling in these farms showing only a proportion of the genotypic richness (**Figure 2D**). Rarefaction curves related to the health status of the farms suggested that the implementation of the drug-free program was associated with the fostering and persistence of a beneficial *C. perfringens* population for the birds, an affirmation supported by the performance results (**Table 1**; Heuer et al., 2008). Moreover, the conservation of a limited genetic richness among the *C. perfringens* population living in these healthy farms over time might have heightened the capacity of this bacterial community to respond to further selective pressure. The richness observed could also represent the successful establishment of a favorable and well-adapted *C. perfringens* population in this novel and challenging environment, generated by the implementation of the drug-free program (Rius and Darling, 2014). The presence of multiple *C. perfringens* clones in healthy birds and healthy flocks has also been reported by other authors (Engstrom et al., 2003; Nauerby et al., 2003; Barbara et al., 2008; Chalmers et al., 2008). The marked reduction in *C. perfringens* species richness observed at the end of the study in farms that experienced recurring clinical outbreaks of NE is informative (**Figure 2B**). Indeed, as previously proposed, birds living in NE-affected farms may have acquired the virulent clone from their environment, potentially contaminated following earlier episodes of subclinical or clinical NE (Palliyeguru and Rose, 2014). Accordingly, given the proper conditions engendered by antimicrobial withdrawal, clinical NE outbreaks occurred in these farms, further increasing the available population of disease-causing strains (Cooper and Songer, 2009). This is also in line with the assumption stating that in a finite population, under specific conditions, a clone that is better adapted and able to use the niche’s resources more appropriately can evolve toward a population in which all organisms are direct descendants of this fitter and favored ancestor (Doolittle, 2012; Maharjan et al., 2012). As a clinical NE outbreak is usually related to the selective multiplication of one or few virulent clones (Sawires and Songer, 2006; Barbara et al., 2008; Timbermont et al., 2009), we propose that, in diseased farms, the *C. perfringens* population was gradually replaced by the clonal expansion of some virulent strains. A similar expansion of a few virulent clones has bene reported by others (Sawires and Songer, 2006; Barbara et al., 2008; Timbermont et al., 2009). Again, it is relevant to mention that antibiotic therapy with penicillin was used to control severe NE outbreaks and avoid further NE-associated complications in two of the eleven (one treatment on each affected farm) NE-affected flocks considered in this study. Though no *C. perfringens* strains from these treated flocks were included in the current study, this treatment might also have, to a limited extent, contributed to the observed decrease in *C. perfringens* genotypic richness by selectively eliminating specific sensitive clones. Our sampling procedure most likely included these virulent clones, as indicated by the loss of species richness over time, as well as the trend toward a more rapidly plateauing curve observed for these diseased farms at the end of the experiment (**Figure 2C**). However, even though rarefaction curves show a loss of species richness over time for NE-affected farms, patterns obtained using a PFGE typing approach indicate that these virulent clones continue to undergo recombination, confirming the dynamic nature of this bacterial species, as previously described (Lacey et al., 2016; Parent et al., 2016). The number of genotypes identified for *C. perfringens* strains obtained from the final sampling in our study is also in line with the literature reporting an increasing *C. perfringens* population richness following a NE outbreak (Nauerby et al., 2003; Barbara et al., 2008; Chalmers et al., 2008).

Some factors might have influenced the ability of these virulent clones to colonize and persist in the NE-affected farms of our study. One explanation could come from the toxinotyping results. It has been reported that *C. perfringens* strains associated with NE outbreaks would not only possess a chromosomal genetic background conferring them a selective advantage over the commensal strains (Chalmers et al., 2008; Hibberd et al., 2011; Lepp et al., 2013; Lacey et al., 2016), but that these strains would also harbor many plasmid-borne virulence genes organized in pathogenicity loci (Lepp et al., 2010; Lacey et al., 2016). Within these loci, some genes encoding toxins, antibiotic resistance, and adhesion factors might preferentially contribute to the ability of the virulent strains to persist over time and to colonize the birds (Lacey et al., 2016; Prescott et al., 2016; Zhou et al., 2017). Indeed, some genes found in these strains could be associated with a better adaptation to an environment where the disease is raging and where they will find the conditions to undergo positive selection and multiplication (Sawires and Songer, 2006; Prescott et al., 2016). The increase in toxin gene carriage observed in the *C. perfringens* population found in NE-affected farms of our study could also be partially explained by the chromosomal background of these virulent strains, providing them with a heightened capacity of exchanging and carrying plasmids. Among the relevant virulence genes, *netB*, which plays a critical role in NE pathogenesis, and *cpb2* are located on those plasmids found in the virulent strains (Lepp et al., 2010; Parreira et al., 2012; Prescott et al., 2016). Surprisingly, in healthy farms, the proportion of strains carrying the *netB* gene at the beginning and at the end of the study was considerable. Though this situation has already been reported (Abildgaard et al., 2010), we would have expected to see this higher prevalence of strains harboring these genes in diseased farms. This result highlights the fact that the sole presence of *netB* is not sufficient to cause clinical disease in a broiler flock (Zhou et al., 2017). The absence of clinical NE in these healthy farms during the study could explain why we did not see any increase in the number of *C. perfringens* strains harboring these two genes at the end of the experiment. Interestingly, in diseased farms, the proportion of *C. perfringens* strains carrying *cpb2* and *netB* genes underwent a significant increase during the field study. Two hypothesis can be proposed to explain this increased frequency in *netB* and *cpb2* harboring strains in the diseased farms. The selective multiplication and amplification over time of dominant clones harboring these genes may have contributed to the observed increase, as well as to the reduced genotypic richness noted for NE-affected farms (**Figure 2A**). Since *cpb2* and *netB* genes reside on mobile genetic elements, we cannot exclude the possibility of an increased horizontal gene transfer between the different variants of *C. perfringens* colonizing the gut of NE-affected birds, a well-recognized ability of *C. perfringens* strains in laboratory conditions (Bannam et al., 2011; Stecher et al., 2012; Lacey et al., 2016). Our results are consistent with those of previous studies reporting the important role of plasmid DNA conjugative transfer in antibiotic resistance and virulence gene transfer in influencing *C. perfringens* adaptation (Wisniewski et al., 2015). However, conducive conditions and proper chromosomal background seem to be essential to promote an optimal horizontal transfer in these NE-causing strains. Since we did not see this upward trend in the frequency of strains harboring more virulence genes, such virulent strains might not have been present or may not have encountered the necessarily favorable conditions for NE development in healthy farms (Lepp et al., 2010; Lacey et al., 2016). Moreover, even if both *cpb-2* and *netB* toxin genes were present at relatively high frequencies in healthy farms at the start of the experiment, no clinical necrotic enteritis outbreak was observed throughout the duration of the study in these farms. The increase observed in the final proportion of strains harboring virulence genes only in NE-affected farms further demonstrates a significant positive selection of virulent *C. perfringens* clones in diseased farms. Since all participating farms were standardized and because the drug-free program was applied in a stringent and uniform way between farms, we can conclude that other contributing factors might have been involved (Gholamiandekhordi et al., 2006; Keyburn et al., 2008; Martin and Smyth, 2009; Brady et al., 2010). The different *C. perfringens* populations living in these farms probably had a role to play, not only in the occurrence of NE at the beginning of the field study, but also in the recurrent nature of these outbreaks (Barbara et al., 2008; Cooper and Songer, 2009; Lepp et al., 2010; Shojadoost et al., 2012).

Our findings show that commercial broiler chicken farms participating in a drug-free program can show significantly different profiles according to clinical NE. These findings can be explained in multiple ways. Among these, comparative genomics realized between virulent and commensal strains of *C. perfringens* recovered from the participating farms could help explain why clinical necrotic enteritis was not observed in healthy farms, despite the presence of strains harboring toxin genes (Lepp et al., 2010, 2013; Parreira et al., 2012). The presence of a specific and beneficial intestinal microflora in birds could also have contributed to this healthy profile and would deserve a more comprehensive assessment (Torok et al., 2011; Singh et al., 2014; Stanley et al., 2014). Ultimately, a resampling of the *C. perfringens* populations living in healthy and diseased participating farms after the reintroduction of antimicrobials could help us to better assess the evolution of these populations under different selective environments.

## Author Contributions

Conception or design of the work; or the acquisition, analysis, or interpretation of data for the work: M-LG, JA, GP, SQ, AL, and MB. Drafting the work or revising it critically for important intellectual content: M-LG, GP, JA, SQ, MB, and AL. Final approval of the version to be published: SQ, JA, GP, AL, MB, and M-LG. Agreement to be accountable for all aspects of the work in ensuring that questions related to the accuracy or integrity of any part of the work are appropriately investigated and resolved: M-LG, JA, GP, SQ, AL, and MB.

## Conflict of Interest Statement

The authors declare that the research was conducted in the absence of any commercial or financial relationships that could be construed as a potential conflict of interest.
